# Development of support vector machine-based model and comparative analysis with artificial neural network for modeling the plant tissue culture procedures: effect of plant growth regulators on somatic embryogenesis of chrysanthemum, as a case study

**DOI:** 10.1186/s13007-020-00655-9

**Published:** 2020-08-13

**Authors:** Mohsen Hesami, Roohangiz Naderi, Masoud Tohidfar, Mohsen Yoosefzadeh-Najafabadi

**Affiliations:** 1 Department of Plant Agriculture, University of Guelph, Guelph, ON Canada; 2 Department of Horticultural Science, Faculty of Agriculture, University of Tehran, Karaj, Iran; 3 Department of Plant Biotechnology, Faculty of Science and Biotechnology, Shahid Beheshti University, G.C., Tehran, Iran

**Keywords:** Artificial intelligence, Support vector regression, Multi-objective optimization algorithm, Machine learning algorithms, Multilayer perceptron, Somatic embryogenesis, Chrysanthemum, Nitric oxide

## Abstract

**Background:**

Optimizing the somatic embryogenesis protocol can be considered as the first and foremost step in successful gene transformation studies. However, it is usually difficult to achieve an optimized embryogenesis protocol due to the cost and time-consuming as well as the complexity of this process. Therefore, it is necessary to use a novel computational approach, such as machine learning algorithms for this aim. In the present study, two machine learning algorithms, including Multilayer Perceptron (MLP) as an artificial neural network (ANN) and support vector regression (SVR), were employed to model somatic embryogenesis of chrysanthemum, as a case study, and compare their prediction accuracy.

**Results:**

The results showed that SVR (R^2^ > 0.92) had better performance accuracy than MLP (R^2^ > 0.82). Moreover, the Non-dominated Sorting Genetic Algorithm-II (NSGA-II) was also applied for the optimization of the somatic embryogenesis and the results showed that the highest embryogenesis rate (99.09%) and the maximum number of somatic embryos per explant (56.24) can be obtained from a medium containing 9.10 μM 2,4-dichlorophenoxyacetic acid (2,4-D), 4.70 μM kinetin (KIN), and 18.73 μM sodium nitroprusside (SNP). According to our results, SVR-NSGA-II was able to optimize the chrysanthemum’s somatic embryogenesis accurately.

**Conclusions:**

SVR-NSGA-II can be employed as a reliable and applicable computational methodology in future plant tissue culture studies.

## Background

Chrysanthemum (*Dendranthema *×* grandiflorum*) can be categorized as one of the most economically important ornamental species due to its color and morphological diversity. Moreover, chrysanthemum has been used as a model plant for color modification studies [1–3]. Conventional propagation and breeding approaches are not able to meet the increasing demands of the market for this valuable ornamental plant. Therefore, novel biotechnological methods can be employed in order to satisfy the demands of consumers [1, 3]. Nowadays, in vitro culture methods as biotechnological tools are applied to the rapid multiplication of rare plant genotypes, micropropagation of disease-free plants, production of plant-derived metabolites, and gene transformation [1, 3–5]. To study in vitro functional genomics, somatic embryos have been employed as a potential explant material [2, 3, 6, 7]. Moreover, many studies proved the usefulness of embryogenesis as a comprehensive model in studying plant growth and development [6, 8, 9]. The unique developmental pathway represented by somatic embryogenesis can be categorized in different characteristic events such as cell differentiation, activation of cell division, dedifferentiation of cells and reprogramming of their metabolism, gene expression patterns, and physiology [9]. Thus, efficient somatic embryogenesis protocol can play a conspicuous role in successful chrysanthemum genetic manipulation and regeneration. Using the appropriate type and concentration of plant growth regulators (PGRs) in various combinations could improve the somatic embryogenesis of different plant species and explants [10–12]. Indeed, in vitro embryogenesis is controlled by the balances of exogenous PGRs and concentrations of endogenous phytohormones. The levels of endogenous phytohormones regulate the in vitro explant differentiation and are assumed to be the major variation sources between different genotypes and explants [1, 13–17]. Therefore, optimizing the somatic embryogenesis protocol can be considered as the first and foremost step in successful gene transformation studies. However, it is usually difficult to achieve an optimized embryogenesis protocol because it is a laborious, time-consuming, and complex process. Therefore, it is necessary to use a novel computational approach for addressing this bottleneck.

In vitro culture consists of highly complex and nonlinear processes such as dedifferentiation, re-differentiation, or differentiation due to the genetic and environmental factors [18–21]. Therefore, it would be difficult to predict different in vitro culture parameters such as callogenesis rate, embryogenesis rate, and the number of somatic embryos as well as optimize factors involved in these parameters by simple conventional mathematical methods [22–24]. Furthermore, biological processes such as somatic embryogenesis cannot be described as a simple stepwise algorithm, especially when the datasets are highly noisy and complex [25–29]. Therefore, machine learning algorithms can be employed as an efficient and reliable computational methodology to interpret and predict different unpredictable datasets [30–34]. Recently, Multilayer Perceptron (MLP) as one of the common artificial neural networks (ANNs) has been widely employed for modeling and predicting in vitro culture systems such as in vitro sterilization [35, 36], callogenesis [37–39], cell growth and protoplast culture [40, 41], somatic embryogenesis [38, 42, 43], shoot regeneration [25, 44–46], androgenesis [47], hairy root culture [48, 49], and in vitro rooting and acclimatization [31]. MLP is a type of nonlinear computational methods, which can be applied for different aims such as clustering, predicting, and classifying the complex systems [47, 50]. MLP is able to identify the relationship between output and input variables and recognize the inherent knowledge existent in the datasets without previous physical considerations [29, 51]. This algorithm consists of numerous highly interconnected processing neurons that work in parallel to find a solution for a particular problem. MLP is learned by example, which should be carefully chosen otherwise time is wasted or even in worse scenarios, the model might be working inaccurately [52].

Support vector machines (SVMs), developed by Vapnik [53], are a kind of interesting, powerful, and easy to interpret machine learning algorithms that analyze data and recognize patterns, used for clustering, classification and regression analysis of nonlinear relationships [54]. Some of the advantages of SVMs in comparison with MLP are related to the complexity of the networks; MLP usually implementing very small number of hidden neurons, whereas SVM uses a large number of hidden units. The best advantage of SVMs is the formulation of the learning problem, resulting in the quadratic optimization task [55, 56]. Support Vector Regression (SVR) is a regression version of SVM. Recently, several studies were published regarding SVM-based approaches in solving industrially or chemically important problems [57–59]. However, SVR, unlike MLP, is relatively unknown to scientists in the field of plant tissue culture. Also, there is no comprehensive study to compare MLP with other machine learning algorithms (e.g. SVR) in order to develop an appropriate model for predicting in vitro culture parameters such as callogenesis rate, embryogenesis rate, and number of somatic embryos.

Different studies [25, 28, 30, 31, 34] have widely employed evolutionary optimization algorithms, in particular, genetic algorithm (GA) as a single optimization algorithm to optimize different factors involved in in vitro culture parameters. This common single-objective optimization algorithm offers merit points over more conventional optimization methods [60, 61]. Also, GA has the benefit that it does not need initial estimates for the decision variables. However, GA can be just employed for a single objective function. On the other hand, the Non-dominated Sorting Genetic Algorithm (NSGA) developed by Srinivas and Deb [62] has been successfully employed to optimize many multi-objective variables. However, the main disadvantage of NSGA has been the lack of elitism, the requirements for specifying sharing parameters, and its high computational complexity of non-dominated sorting [60]. NSGA-II is the improved version of NSGA, which has a better incorporates elitism, sorting algorithm, and no sharing parameter requires to be chosen a priori [35]. Therefore, the elitist NSGA-II can be utilized for multi-objective optimization with two, three, or more objective functions [7]. Recently, NSGA-II has been successfully applied to optimize shoot regeneration rate, number of shoots, and callus weight, simultaneously [33].

In the current study, SVR has been employed to predict the somatic embryogenesis parameters, including callogenesis rate, embryogenesis rate, and the number of somatic embryos of chrysanthemum. The developed SVR-based model was compared with MLP in terms of statistical performance parameters to find the most suitable model for modeling and predicting in vitro culture systems. Furthermore, NSGA-II was linked to the best model to find the optimal level of PGRs for somatic embryogenesis. According to the best of our knowledge, this study is the first report of the application of SVR in the field of plant tissue culture.

## Results

### Effects of PGRs on somatic embryogenesis

Although several investigations have focused on the impact of auxins and cytokinins concentrations in chrysanthemum embryogenesis, there is a lack of study on the influence of auxins, cytokinins, nitric oxide, and their interactions. The PGRs are essential factors in plant tissue culture processes that are remarkably impacted the somatic embryogenesis. The current study was determined the effects of 2,4-dichlorophenoxyacetic acid (2,4-D), kinetin (KIN), sodium nitroprusside (SNP), and their interactions on callogenesis rate (%), number of somatic embryos per explant, and embryogenesis rate (%) of chrysanthemum.

The results of this study showed that leaf explants in the medium containing both 2,4-D and KIN led to both callogenesis and embryogenesis. On the other hand, the medium without PGR was not able to produce calli and embryos. After two and three weeks from culturing, the cut ends of the leaf segments produced calli and embryos, respectively. According to Table 1, high embryogenesis rate and the number of somatic embryos per explant were achieved by using SNP along with 2,4-D and KIN, which is higher than that produced by the media without SNP. Also, the highest callogenesis rate (100%), embryogenesis rate (100%), and the number of somatic embryos per explant (57.8) were observed in the combination of 9.09 μM 2,4-D and 4.65 μM BAP along with 20 μM SNP (Table 1).

**Table 1:** Effects of 2,4-D, KIN, and SNP on callogenesis rate, number of somatic embryos, and embryogenesis rate of chrysanthemum of chrysanthemum

2,4-D (μM)	Kin (μM)	SNP (μM)	Callogenesis rate (%)	Embryogenesis rate (%)	Embryo number
0	0	0	0.00 ± 0.00	0.00 ± 0.00	0.00 ± 0.00
4.54	0	0	84.44 ± 5.56	0.00 ± 0.00	0.00 ± 0.00
9.09	0	0	93.33 ± 3.33	0.00 ± 0.00	0.00 ± 0.00
13.63	0	0	100.00 ± 0.00	0.00 ± 0.00	0.00 ± 0.00
0	4.65	0	0.00 ± 0.00	0.00 ± 0.00	0.00 ± 0.00
4.54	4.65	0	80.00 ± 4.71	48.89 ± 5.88	4.48 ± 0.34
9.09	4.65	0	100.00 ± 0.00	100.00 ± 0.00	31.71 ± 0.74
13.63	4.65	0	100.00 ± 0.00	71.11 ± 5.88	9.02 ± 0.34
0	9.29	0	22.22 ± 7.78	0.00 ± 0.00	0.00 ± 0.00
4.54	9.29	0	91.11 ± 4.84	73.33 ± 5.77	7.69 ± 0.24
9.09	9.29	0	100.00 ± 0.00	100.00 ± 0.00	21.73 ± 0.44
13.63	9.29	0	100.00 ± 0.00	100.00 ± 0.00	4.23 ± 0.30
0	13.94	0	24.44 ± 8.01	0.00 ± 0.00	0.00 ± 0.00
4.54	13.94	0	97.78 ± 2.22	60.00 ± 6.67	6.93 ± 0.24
9.09	13.94	0	100.00 ± 0.00	86.67 ± 4.71	13.01 ± 0.36
13.63	13.94	0	100.00 ± 0.00	100.00 ± 0.00	4.06 ± 0.24
0	0	10	0.00 ± 0.00	0.00 ± 0.00	0.00 ± 0.00
4.54	0	10	88.89 ± 4.84	0.00 ± 0.00	0.00 ± 0.00
9.09	0	10	95.56 ± 2.94	0.00 ± 0.00	0.00 ± 0.00
13.63	0	10	100.00 ± 0.00	0.00 ± 0.00	0.00 ± 0.00
0	4.65	10	0.00 ± 0.00	0.00 ± 0.00	0.00 ± 0.00
4.54	4.65	10	91.11 ± 4.84	62.22 ± 7.03	5.96 ± 0.39
9.09	4.65	10	100.00 ± 0.00	100.00 ± 0.00	35.60 ± 0.69
13.63	4.65	10	100.00 ± 0.00	86.67 ± 4.71	9.87 ± 0.36
0	9.29	10	31.11 ± 6.76	0.00 ± 0.00	0.00 ± 0.00
4.54	9.29	10	95.56 ± 4.44	86.67 ± 4.71	8.82 ± 0.29
9.09	9.29	10	100.00 ± 0.00	100.00 ± 0.00	25.86 ± 0.63
13.63	9.29	10	100.00 ± 0.00	100.00 ± 0.00	5.51 ± 0.26
0	13.94	10	33.33 ± 7.45	0.00 ± 0.00	0.00 ± 0.00
4.54	13.94	10	100.00 ± 0.00	75.56 ± 4.44	7.77 ± 0.20
9.09	13.94	10	100.00 ± 0.00	100.00 ± 0.00	16.79 ± 0.37
13.63	13.94	10	100.00 ± 0.00	100.00 ± 0.00	5.28 ± 0.19
0	0	20	0.00 ± 0.00	0.00 ± 0.00	0.00 ± 0.00
4.54	0	20	95.56 ± 2.94	2.22 ± 2.22	0.22 ± 0.22
9.09	0	20	100.00 ± 0.00	4.44 ± 2.94	0.33 ± 0.24
13.63	0	20	100.00 ± 0.00	0.00 ± 0.00	0.00 ± 0.00
0	4.65	20	13.33 ± 7.45	8.89 ± 4.84	0.73 ± 0.37
4.54	4.65	20	100.00 ± 0.00	84.44 ± 5.56	9.56 ± 0.21
9.09	4.65	20	100.00 ± 0.00	100.00 ± 0.00	57.80 ± 0.21
13.63	4.65	20	100.00 ± 0.00	100.00 ± 0.00	17.07 ± 0.29
0	9.29	20	46.67 ± 5.77	13.33 ± 4.71	0.81 ± 0.30
4.54	9.29	20	100.00 ± 0.00	100.00 ± 0.00	11.64 ± 0.19
9.09	9.29	20	100.00 ± 0.00	100.00 ± 0.00	29.08 ± 0.26
13.63	9.29	20	100.00 ± 0.00	100.00 ± 0.00	7.38 ± 0.20
0	13.94	20	57.78 ± 7.03	17.78 ± 5.21	0.78 ± 0.22
4.54	13.94	20	100.00 ± 0.00	95.56 ± 2.94	11.38 ± 0.26
9.09	13.94	20	100.00 ± 0.00	100.00 ± 0.00	25.63 ± 0.42
13.63	13.94	20	100.00 ± 0.00	100.00 ± 0.00	8.60 ± 0.34
0	0	40	0.00 ± 0.00	0.00 ± 0.00	0.00 ± 0.00
4.54	0	40	97.78 ± 2.22	0.00 ± 0.00	0.00 ± 0.00
9.09	0	40	100.00 ± 0.00	2.22 ± 2.22	0.22 ± 0.22
13.63	0	40	100.00 ± 0.00	0.00 ± 0.00	0.00 ± 0.00
0	4.65	40	17.78 ± 6.19	8.89 ± 3.51	0.44 ± 0.18
4.54	4.65	40	100.00 ± 0.00	77.78 ± 5.21	8.06 ± 0.13
9.09	4.65	40	100.00 ± 0.00	100.00 ± 0.00	45.77 ± 0.33
13.63	4.65	40	100.00 ± 0.00	100.00 ± 0.00	14.54 ± 0.20
0	9.29	40	31.11 ± 4.84	11.11 ± 4.84	0.44 ± 0.18
4.54	9.29	40	100.00 ± 0.00	100.00 ± 0.00	8.83 ± 0.18
9.09	9.29	40	100.00 ± 0.00	100.00 ± 0.00	24.74 ± 0.18
13.63	9.29	40	100.00 ± 0.00	100.00 ± 0.00	6.58 ± 0.17
0	13.94	40	68.89 ± 5.88	11.11 ± 3.51	0.56 ± 0.18
4.54	13.94	40	100.00 ± 0.00	93.33 ± 3.33	10.60 ± 0.14
9.09	13.94	40	100.00 ± 0.00	100.00 ± 0.00	21.32 ± 0.28
13.63	13.94	40	100.00 ± 0.00	91.11 ± 3.51	7.59 ± 0.18

Values in each column represent mean ± standard error

### SVR modeling and evaluation

SVR was used for modeling the three target variables (callogenesis rate, embryogenesis rate, and the number of somatic embryos) based on three input variables, including 2,4-D, KIN, and SNP.

**Fig. 1:**
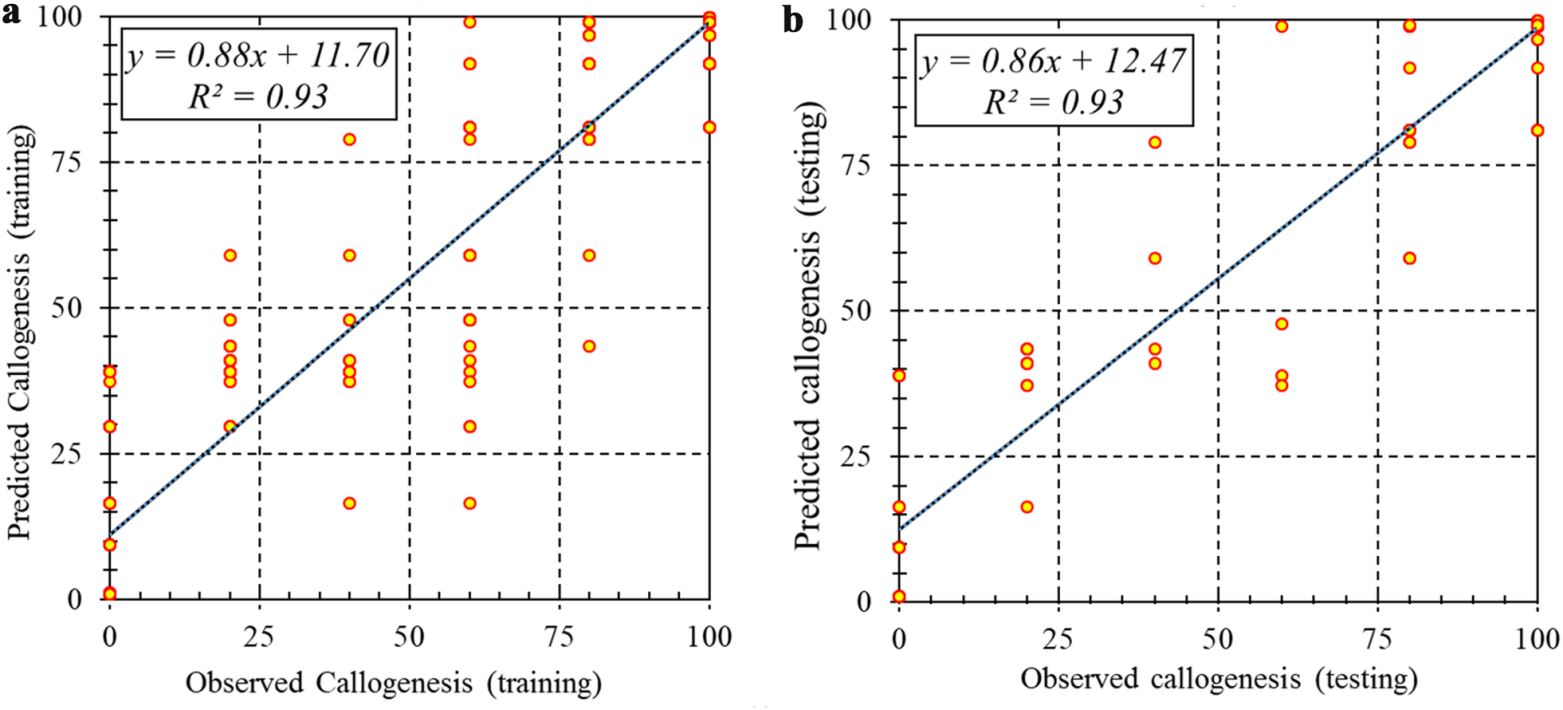
Scatter plot of model predicted vs. observed data of chrysanthemum callogenesis rate for PGRs adjustment obtained by SVR model. **a** Training set (n = 432). **b** Testing set (n = 144)

**Fig. 2:**
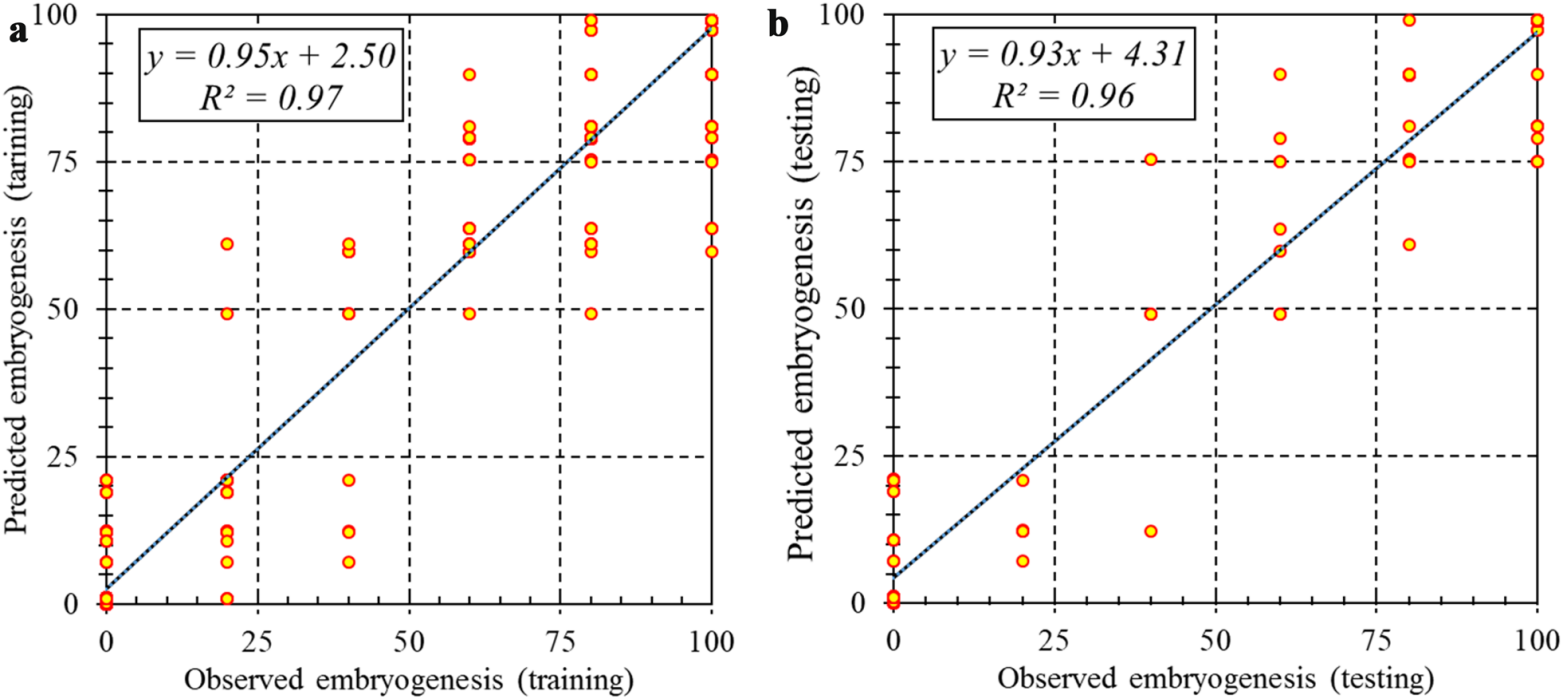
Scatter plot of model predicted vs. observed values of chrysanthemum embryogenesis rate for PGRs adjustment obtained by SVR model. **a** Training set (n = 432). **b** Testing set (n = 144)

**Fig. 3:**
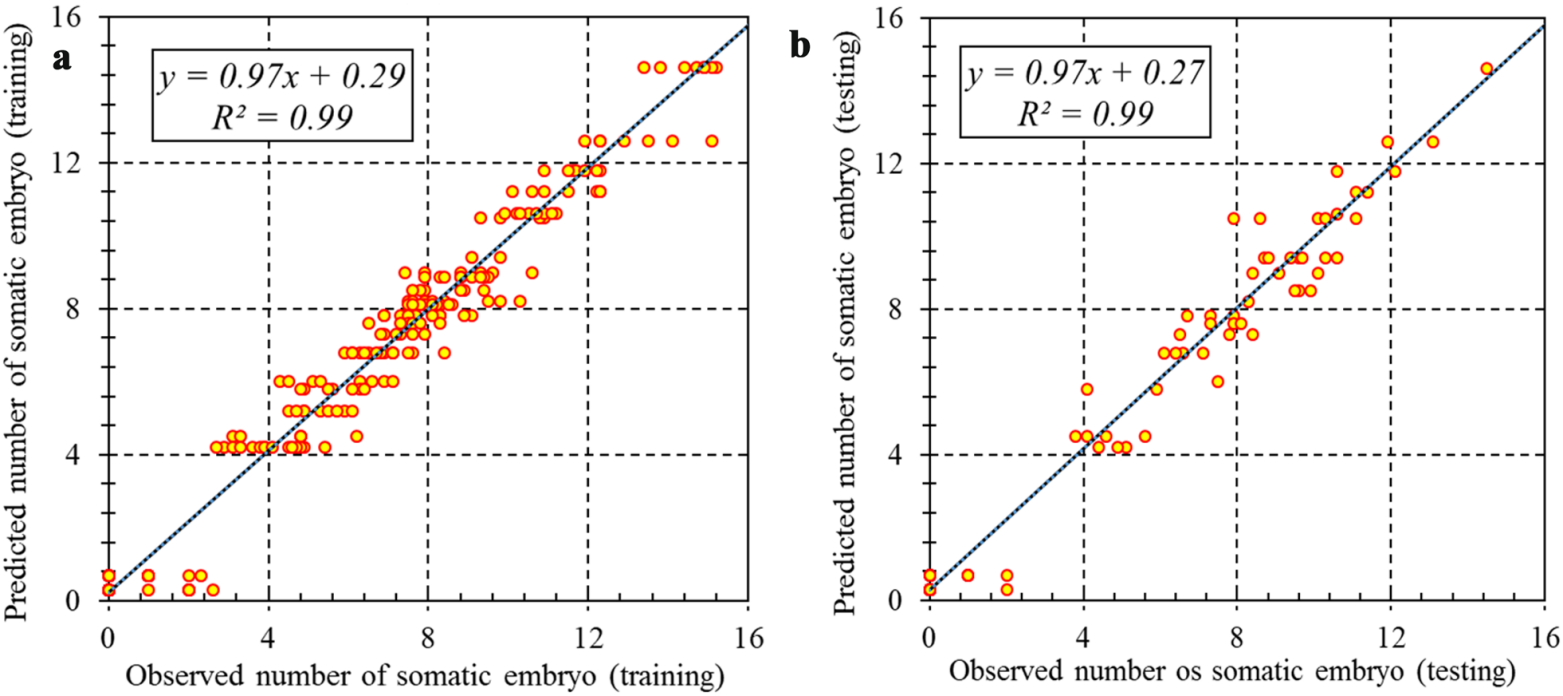
Scatter plot of model predicted vs. observed values of number of chrysanthemum somatic embryos for PGRs adjustment obtained by SVR model. **a** Training set (n = 432). **b** Testing set (n = 144)

Two machine learning algorithms, including MLP and SVR were used for modeling and predicting target variables. R^2^, RMSE, and MAE of each developed model were presented in Table 2. Comparative analysis of MLP and SVR (Table 2) showed that SVR was more accurate than MLP in all studied parameters in somatic embryogenesis in both training and testing sets. As can be seen in Figs. 1, 2 and 3, the regression lines demonstrated that a good fit correlation between the predicted and observed data of callogenesis rate, embryogenesis rate, and the number of somatic embryos for both the training and testing set. R^2^, RMSE, and MAE of SVR vs. MLP for callogenesis rate were 0.93 vs. 0.89, 9.82 vs.10.03, and 1.33 vs. 1.64 during the training set, and 0.93 vs. 0. 82, 10.67 vs. 15.40, and 1.87 vs. 2.01 during testing set, respectively (Table 2). R^2^, RMSE, and MAE of SVR vs. MLP for embryogenesis rate were 0.97 vs. 0.93, 8.47 10.00, and 0.071 vs. 1.75 during the training set, and 0.96 vs. 0. 90, 9.71 vs. 13.75, and 0.55 vs. 1.91 during testing set, respectively. Also, the performance parameters for the training set for the number of somatic embryos were R^2^ = 0.99 and 0.96, RMSE = 0.81 and 1.64, MAE = 0.02 and 0.06 for SVR and MLP, respectively, and in the testing data set for the number of somatic embryos were R^2^ = 0.99 and 0.91, RMSE = 0.94 and 2.07, MAE = 0.004 and 0.021 for SVR and MLP, respectively (Table 2).

**Table 2:** Statistics of MLP and SVR models for callogenesis rate, number of somatic embryos, and embryogenesis rate of chrysanthemum in training and testing process

Model	Item	Callogenesis rate		Embryogenesis rate		Embryo number	
		Training	Testing	Training	Testing	Training	Testing
SVR	R^2^	0.928	0.928	0.966	0.956	0.996	0.994
	RMSE	9.822	10.697	8.474	9.715	0.813	0.942
	MAE	1.327	1.871	0.071	0.555	0.018	0.004
MLP	R^2^	0.893	0.824	0.927	0.905	0.961	0.912
	RMSE	10.029	15.403	10.003	13.747	1.645	2.073
	MAE	1.644	2.012	1.746	1.908	0.061	0.021

### Sensitivity analysis of the models

Five hundred seventy-six data points were used to determine the overall variable sensitivity ratio (VSR) for identifying the comparative rank of inputs. The results of the sensitivity analysis were summarized in Table 3. Based on sensitivity analysis, callogenesis rate was more sensitive to 2,4-D, followed by KIN, and SNP (Table 3). Also, as can be seen in Table 3, 2,4-D was the most important factor for both embryogenesis rate and the number of somatic embryos per explant, followed by SNP and KIN.

**Table 3:** Importance of PGRs for callogenesis rate, number of somatic embryos, and embryogenesis rate of chrysanthemum according to sensitivity analysis

Output	Item	2,4-D	KIN	SNP
Callogenesis rate	VSR	4.10	1.94	1.49
	Rank	1	2	3
Embryogenesis rate	VSR	5.86	2.30	5.69
	Rank	1	3	2
Number of somatic embryos	VSR	100.33	98.93	99.04
	Rank	1	3	2

### Model optimization

**Fig. 4:**
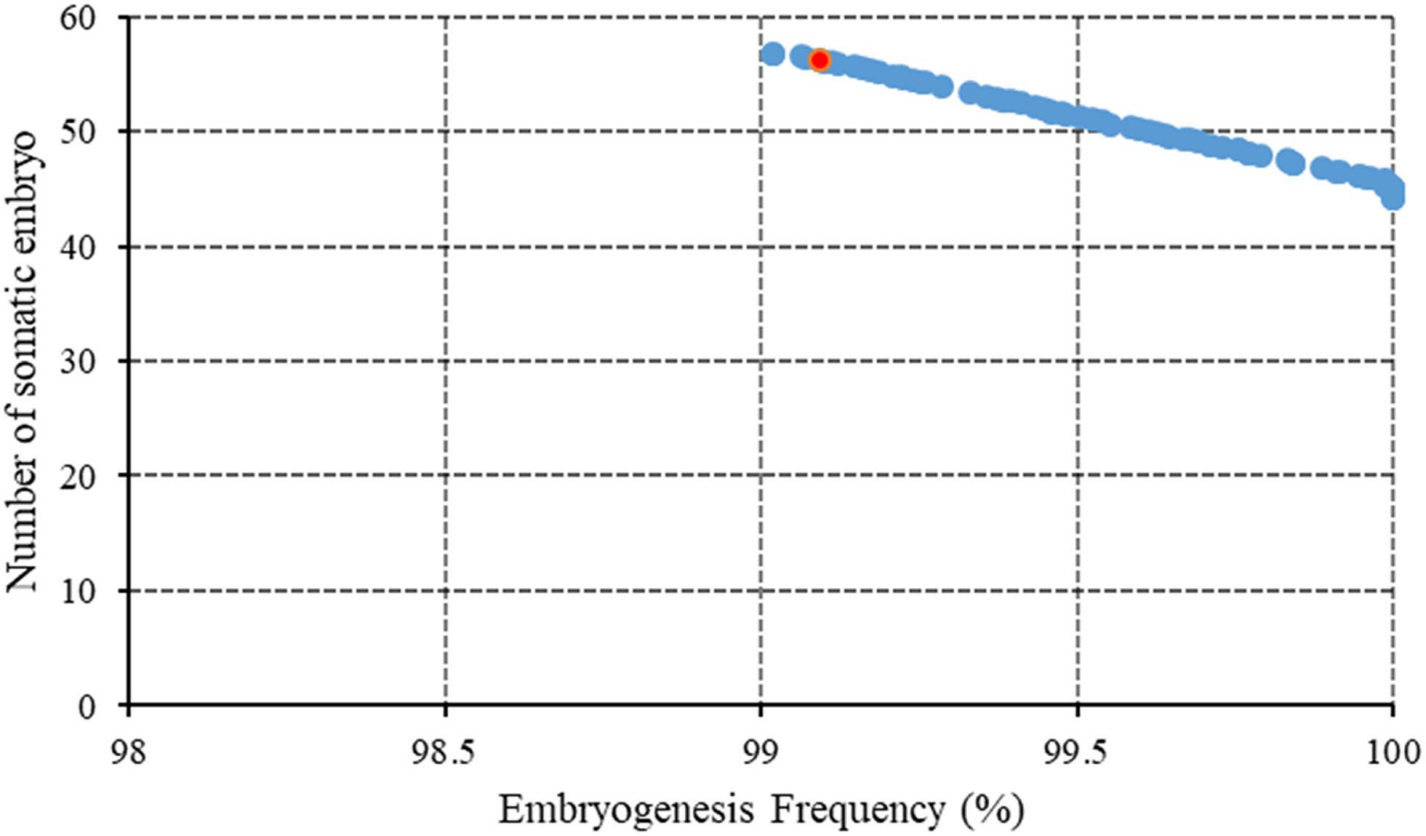
Pareto front obtained by NSGA-II as a multi-objective optimization algorithm for the highest of embryogenesis rate and the maximum number of somatic embryos per explant of chrysanthemum. The ideal point is presented as the red point

NSGA-II was linked to the SVR in order to determine the optimal level of 2,4-D, KIN, and SNP for obtaining the highest embryogenesis rate and the maximum number of somatic embryos per explant. The results of the optimization process were presented in Table 4 and Fig. 4. As can be seen in Table 4, the highest embryogenesis rate (99.09%) and the maximum number of somatic embryos per explant (56.24) can be obtained from a medium containing 9.10 μM 2,4-D, 4.70 μM KIN, and 18.73 μM SNP.

**Table 4:** Optimizing PGRs according to optimization process via SVR-NSGAII for embryo number and embryogenesis rate in chrysanthemum

input variable (μM)			Predicted embryogenesis rate	Predicted embryo number
2,4-D	KIN	SNP		
9.10	4.70	18.73	99.09	56.23

### Validation experiment

According to the validation experiment, the differences between biological validation data and predicted data via SVR-NSGA-II were not significant (Table 5). Indeed, the optimized level of PGRs (9.10 μM 2,4-D, 4.70 μM KIN, and 18.73 μM SNP) led to the highest embryogenesis rate (100%) and the maximum number of somatic embryos per explant (57.86) which is negligibly higher than the predicted result. Therefore, it can be concluded that SVR-NSGA-II can be employed for accurately predicting and optimizing plant tissue culture processes.

**Table 5:** Experimental validation of the predicted-optimized result via SVR-NSGA-II for embryo number and embryogenesis rate of chrysanthemum

Treatment	Embryogenesis rate (%)	Embryo number
9.1 μM 2,4-D + 4.7 μM KIN + 18.73 μM SNP	100 ± 0.00	57.86 ± 0.42

## Discussion

Being successful in in vitro somatic embryogenesis depends on different factors such as the composition of the medium, gelling agents, light and temperature conditions, and the application of specific combinations of PGRs [1, 13–16, 63]. However, optimizing these factors is time and cost consuming. Also, somatic embryogenesis is a highly complex and nonlinear process. Therefore, there is a dire need to employ robust nonlinear computational methods for optimizing embryogenesis parameters. The efficiency of a good statistical approach depends on the neat understanding of the variable structure, experimental design, and using the appropriate model [64]. One of the most important primary requirements to identify suitable statistical approaches is comprehending the type of data [65]. Variables can be clustered into two groups, including quantitative (continuous and discrete) and qualitative (ordinal and nominal). Names with two or more classes without a hierarchical order are categorized as nominal variables, while ordinal data have distinct order (level X is more intense than level Y) [65, 66]. Counts that include integers are classified as discrete data, while measurements along a continuum, which could be included smaller fractions, are categorized as continuous variables [67]. Plant tissue culture data can be categorized as ordinal (callus quality rated as weak, moderate, and good), nominal (callus types such as embryogenic and non-embryogenic callus), continuous (embryogenesis rate), and discrete (number of somatic embryos). Traditional linear methods such as regression and ANOVA must be just applied with continuous variables that demonstrate a linear relationship between the explanatory and dependent variables [52, 68]. On the other hand, in vitro culture systems are considered as complex biological systems that multiple factors can affect the system in nonlinear ways. Hence, the conventional computational approaches are not appropriate for analyzing plant tissue culture data [65]. Recently, different machine learning algorithms such as neural networks [34, 46, 47], fuzzy logic [7, 69], and decision trees [70, 71] have been successfully employed for predicting and optimizing various in vitro culture processes. Many studies [35, 44, 46, 72] used MLP to predict the optimal in vitro conditions for different plant tissue culture systems. However, they only applied the MLP model and did not compare this common algorithm with other models. Another promising computational method not previously employed in in vitro data analyses is the SVR. In the current study, MLP and SVR, for the first time, were used to develop a suitable model for chrysanthemum somatic embryogenesis and compare their prediction accuracy. According to our results, SVR had more accuracy than MLP for modeling and predicting the system. Although there is no report regarding the application of SVR in plant tissue culture, in line with our results, comparative studies in other fields revealed the better performance of SVR in comparison to ANNs such as MLP [57–59]. On the other hand, one of the weaknesses of using machine learning algorithms is that it is hard to obtain an optimized solution [52]. To tackle this problem, several studies [25, 28, 30, 31, 34] used GA to optimize in vitro culture conditions. However, plant tissue culture consists of different functions that sometimes they show conflict interaction. Hence, GA, as a single objective function, cannot optimize multi-objective function [7]. Therefore, it is necessary to employ multi-objective optimization algorithms such as NSGA-II. In the current study, NSGA-II was linked to SVR as the most suitable model for the optimization process. After predicting and optimizing somatic embryogenesis via SVR-NSGA-II, the predicted-optimized results were experimentally tested. Based on our results, SVR-NSGA-II can be considered as an efficient computational methodology for predicting and optimizing different plant tissue culture systems.

The results of the sensitivity analysis showed that 2,4-D is the most important component in the somatic embryogenesis followed by SNP as a donor nitric oxide (NO), and KIN. In line with our results, after several years of molecular and biological somatic embryogenesis studies, it has been shown that 2,4-D is the most important signaling in somatic embryogenesis followed by NO and cytokinin signaling [73]. The type and concentration of PGRs play a pivotal role in somatic embryogenesis. Several studies [1, 14, 74] have elucidated that among tested auxins, 2,4-D as one of the synthetic auxins resulted in the maximum somatic embryogenesis in chrysanthemum. In addition, kinetin, as a cytokinin, promotes somatic embryogenesis in the chrysanthemum [75, 76]. For instance, Shinoyama et al. [75] reported that the maximum number of the somatic embryos (21.3 ± 1.2) was obtained from 2 mg/l 2,4-D along with 1 mg/l kinetin. Nitric oxide is known as a messenger molecule regulating plant development and a ubiquitous bioactive molecule mainly contributed to various plant developmental processes such as fruit ripening, flowering, organ senescence, and germination [73]. This molecule has recently been characterized as one of the phytohormones [77]. The exterior usage of nitric oxide might improve the tolerance of plants under various stresses such as temperature, heavy metals, ultraviolet radiation, drought, and salinity [78–80]. The activation rate of nitric oxide has been evaluated by the exterior usage of sodium nitroprusside (SNP) instead of using NO gas directly because of some technical difficulties [81]. In recent years, nitric oxide gets involved in developing in vitro plant propagation [82]. Ötvös et al. [9] demonstrated that despite NO does not affect cell cycle progression in plant tissue culture, it may have a close relation with auxins linking the adjust of cell division to differentiation. Plants have significant developmental plasticity in comparison with animals. During the de- differentiation process, somatic plant cells can repossess the ability to divide and ‘de-differentiated’ plant cells can ‘re-differentiate’ into whole plants under appropriate conditions. Ötvös et al. [9] reported that NO accompany with auxin can play a significant role in the embryogenesis of leaf protoplast-derived cells. In the absence of auxin, SNP could not induce the protoplast-derived cells division. Also, the alternative response of protoplast-derived cells to various concentrations of external auxin in the presence of SNP or L-NMMA may show that NO can alter the sensitivity of the cells to auxin and involved in intermediary of the auxins role during these processes [8].

Furthermore, NO and auxins were suggested to share similar steps in signal transduction pathways caused to root formation and root elongation [73]. In addition to affecting the dividing cells frequency, SNP and L-NMMA have a massive impact on the pathway of auxin concentration-dependent development of leaf protoplast obtained from cells [73]. It previously indicated that these cells could develop into elongated cells or small, vacuolized, and isodiametric cells with dense cytoplasm showing embryogenic competence [83–85]. Although the formation of embryo-genic-type cells can be obtained at the high concentration of auxins (5–10 μM 2,4-D), by using SNP, this type of cell can be achieved at a low concentration of 2,4-D [9]. Somatic embryo formation can be obtained by the high-level expression of the MsSERK1 gene as well as the development of the cells [86] so this fact proved the usage of SNP in altering the pathway of the auxin-treated cells. SERK gene expression is usually applied as a marker of embryogenic potential [73] despite its up-regulated expression. This was also accompanying with auxin-promoted root formation [86] and was recommended to be morphogenic instead of only being an embryogenic marker.

## Conclusion

Recently, MLP has been widely applied for modeling and predicting in vitro culture systems. In the current study, SVR for the first time was applied to model and predict somatic embryogenesis and to compare its accuracy with MLP. Our results showed that the SVR model has better accuracy than MLP for modeling and predicting complex systems such as somatic embryogenesis. Also, SVR-NSGA-II was able to optimize the chrysanthemum’s somatic embryogenesis accurately. The results of the sensitivity analysis showed that 2,4-D is the most important component in the somatic embryogenesis followed by SNP as a donor nitric oxide (NO), and KIN. Interestingly, after several years of molecular and biological somatic embryogenesis studies, it has been shown that 2,4-D is the most important signaling in somatic embryogenesis followed by NO and cytokinin signaling. These results demonstrate that SVR-NSGA-II can open a reliable and accurate window to a comprehensive study of the plant’s biological processes. It would be recommended to compare SVR with the current machine-learning methods (e.g., Random Forest, Gradient Boosting), to allow a more thorough appreciation of the relative merit of SVM applied to the presented problem.

## Methods

### Plant material, media, and culture condition

In this study, leaf explants of chrysanthemum ‘Hornbill Dark’ were selected for in vitro somatic embryogenesis study. To primary disinfect, the explants were washed for 20 min with tap water. Then, further steps were performed under a laminar airflow cabinet. Subsequently, the explants were soaked with 70% ethanol for 40 s and then washed with sterilized distilled water for 3 min. Afterward, the explants dipped in 1.5% (v/v) NaOCl solution for 15 min. Subsequently, the explants were washed with sterilized distilled water for 5 min three times. The basal medium in this study was Murashige and Skoog [87] (MS) medium consisted of 3% sucrose, 0.7% agar, and 100 mg/l Myo-inositol. Also, the pH of the medium by using 1 and/or 0.1 N NaOH as well as 1 and/or 0.1 N HCl was adjusted to 5.8 before autoclaving for 20 min at 120 ◦C. The explants were cultured in 200-ml culture boxes supplemented with 45 ml basal media. All culture boxes were kept in the growth chamber under 16-h Photoperiod with 50 μmol m^−2^ s^−1^ light intensity at 25 + 2 °C.

### Experimental design

The leaf explants were cultured in the basal medium containing different concentrations of 2,4-D, (0, 4.54, 9.09, and 13.63 μM) Kinetin (KIN) (0, 4.65, 9.29, and 13.94 μM), and sodium nitroprusside (SNP) (0, 10, 20, and 40 μM). The callogenesis rate (Eq. 1), embryogenesis rate (Eq. 2), and the number of somatic embryos were calculated after 6 weeks of culture.

1 $$ {\text{Callogenesis}}\;{\text{ rate}}\; \, \left( \% \right) = \frac{{{\text{Number }}\;{\text{of }}\;{\text{explants }}\;{\text{that }}\;{\text{produce }}\;{\text{callus}}}}{{{\text{Total }}\;{\text{number}}\; {\text{of}}\; {\text{explants}}}} \times 100 $$

2 $$ {\text{Embryogenesis }}\;{\text{rate}}\; \, \left( \% \right) = \frac{{{\text{Number}}\; {\text{of }}\;\;{\text{explants }}\;{\text{that }}\;{\text{produce}}\; {\text{embryo}}}}{{{\text{Total }}\;{\text{number }}\;{\text{of }}\;{\text{explants}}}} \times 100 $$

The somatic embryogenesis experiments were conducted based on a randomized complete block design (RCBD) with a factorial arrangement with a total of 64 treatments with nine replications per treatment, and each replication consisted of five leaf explants.

### Modeling procedures

The input variables were 2,4-dichlorophenoxyacetic acid (2,4-D), kinetin (KIN), sodium nitroprusside (SNP). The target variables were callogenesis rate, embryogenesis rate, and the number of the somatic embryos per explant. Before modeling, the datasets were scaled between 0 and 1 to ensure that all variables receive equal attention during the training process. In the current study, two types of machine learning algorithms, including MLP and SVR, were employed to model somatic embryogenesis of chrysanthemum. To train and test each model, 70 and 30% of the data lines were randomly selected, respectively.

### Multilayer perceptron (MLP) model

The MLP, as one of the common ANNs, consists of three layers, including input, hidden, and output. In the present study, this model was employed, according to Hesami et al. [35] procedure. Briefly, in the present investigation, a 3-layer backpropagation network (feed-forward backpropagation) was applied for constructing the MLP model. To determine the optimal weights and bias as well as train the network, a Levenberg–Marquardt algorithm was applied. Also, the hyperbolic tangent sigmoid (tansig) and linear (purelin) activation functions were utilized for hidden and output layers, respectively.

### Support vector regression (SVR) model

**Fig. 5:**
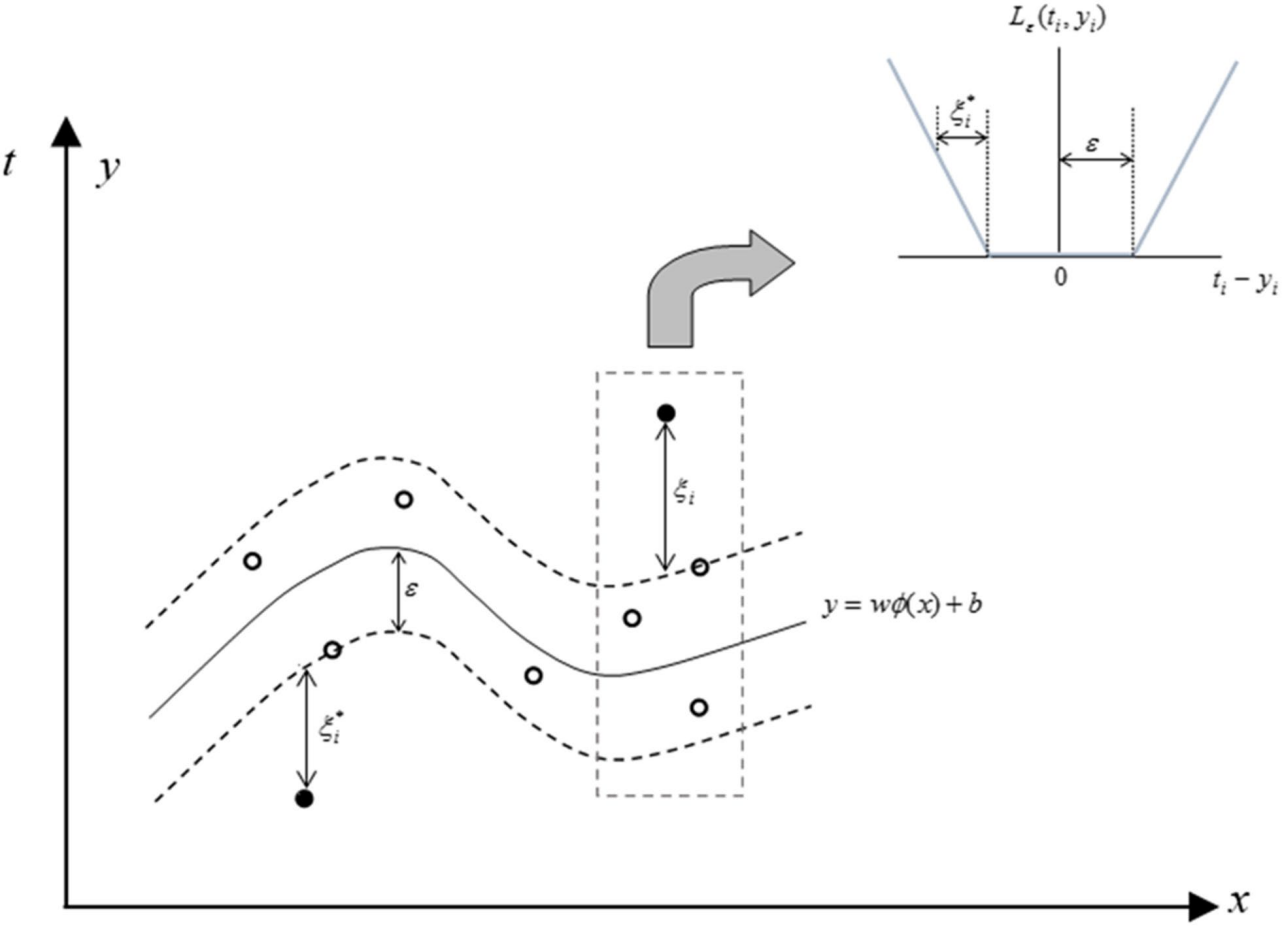
The schematic view of the support vector regression (SVR) model

Support vector machines (SVMs), developed by Vapnik [53], can be used for clustering, classification, and regression analysis of nonlinear relationships [54]. SVR, as a regression version of SVM, was employed in the current study. Considering $\{ (x_{i} ,t_{i} )\}_{i}^{n}$ as a dataset, *x*_*i*_ shows *i*^*th*^ input vector, *t*_*i*_ represents *i*^*th*^ output vector, and *n* equals a total number of observations. The following function used for the SVR estimation:

3 $$ y = w\varphi (x) + b $$

where *w* shows weights, *b* is bias, and $\varphi (x)$ represents the high dimensional feature space, which is non-linearly mapped from the input space *x* and *y* is output value. SVR tried to minimize a loss function, and the main goal is that all the estimated variables are placed between the upper and lower prediction error bounds. Upper and lower prediction error bounds in SVR are $y = w\varphi (x) + b + \varepsilon$ and $y = w\varphi (x) + b - \varepsilon$, respectively. Figure 5 shows a schematic view of SVR. An optimization process was used to find out *w* and *b* coefficients as follows:

4 $$ Min:\;L_{{}} = C\frac{1}{n}\sum\limits_{i = 1}^{n} {L_{\varepsilon } } (t_{i} ,y_{i} ) + \frac{1}{2}w.w^{T} $$

5 $$ L_{\varepsilon } (t_{i} ,y_{i} ) = \left\{ \begin{aligned} |t - y| - \varepsilon \quad \quad |t - y| > \varepsilon \hfill \\ 0\quad \quad \quad \quad \quad \;otherwise \hfill \\ \end{aligned} \right. $$

where, $\varepsilon$, $L_{\varepsilon }$, and *C* represents an acceptable error (tube size), insensitive loss function, and penalty parameter, respectively. Both and *C* are user-prescribed parameters. The dual function of the problem with the application of Lagrange multipliers is as follows:

6 $$ \begin{aligned} & Max\;L_{D} = \sum\limits_{i = 1}^{n} {t_{i} (\alpha_{i} - \alpha_{i}^{*} } ) - \varepsilon \sum\limits_{i = 1}^{n} {(\alpha_{i} } + \alpha_{i}^{*} ) \\ & \;\quad \quad \quad \quad - \frac{1}{2}\sum\limits_{i = 1}^{n} {\sum\limits_{j = 1}^{n} {(\alpha_{i} - \alpha_{i}^{*} )} } (\alpha_{j} - \alpha_{j}^{*} )k(x_{i} ,x_{j} ) \\ & Subjected\;\quad to: \\ & \sum\limits_{i = 1}^{n} {(\alpha_{i} - \alpha_{i}^{*} ) = 0} \\ & 0 \le \alpha_{i} \le C\quad i = 1,2, \ldots ,n \\ & 0 \le \alpha_{i}^{*} \le C\quad i = 1,2, \ldots ,n \\ \end{aligned} $$

After solving the optimization problem, *w* and *b* are determined. The lagrange multipliers with non-zero values were assumed as the supporting vector. Then the SVR can be carried out as follows:

7 $$ y = \sum\limits_{i = 1}^{n} {(\alpha_{i} - \alpha_{i}^{*} } )k(x,x_{i} ) + b $$

Among the various kernel functions in SVR, radial basis function (RBF) is one of the common kernel functions for nonlinear problems. Therefore, SVR along with RBF kernel function could be presented with three parameters as SVR (y, C, Ɛ).

### Performance measures

To assess and compare the accuracy of mentioned models, three following performance measures including *R*^*2*^ (coefficient of determination), Root Mean Square Error (RMSE), and Mean Absolute Error (MAE) were used:

8 $$ R^{2} = \left[ {\frac{{\mathop \sum \nolimits_{t = 1}^{T} \left( {y_{t} - \bar{y}} \right)\left( {\hat{y}_{t } - \hat{\bar{y}}} \right)}}{{\sqrt {\mathop \sum \nolimits_{t = 1}^{T} \left( {y_{t } - \bar{y}} \right) }     \sqrt {\mathop \sum \nolimits_{t = 1}^{T} \left( {\hat{y}_{t } - \hat{\bar{y}}} \right)} }}} \right]^{2} $$

9 $$ MAE = 1/n\mathop \sum \limits_{i = 1}^{n} \left| {y_{i} - \hat{y}_{i} } \right| $$

10 $$ RMSE\, = \,\sqrt {\left( {\sum\nolimits_{i = 1}^{n} {\left( {y_{i} - \,\hat{y}_{i} } \right)^{2} } } \right)\,/\,n} $$

where *y*_*t*_, $\bar{y}$, $\hat{y}_{t }$, and *T* are the *t*^th^ observed data, the mean of observed values, the mean of predicted values, and total number of predicted values, respectively. Greater R^2^ and smaller RMSE and MAE indicated better performance of the constructed models.

### Optimization of somatic embryogenesis via NSGA-II

**Fig. 6:**
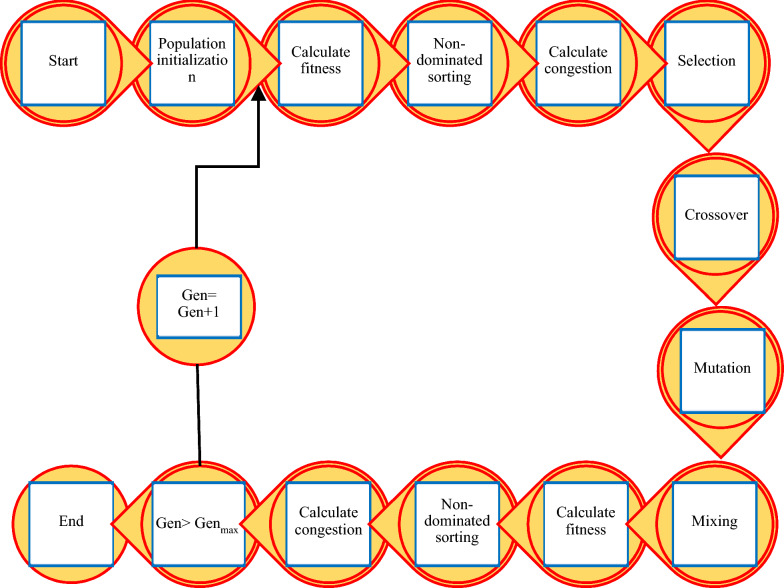
The schematic diagram illustrating optimization process via Non-dominated Sorting Genetic Algorithm-II (NSGA-II)

To identify the optimal levels of inputs (2,4-D, KIN, and SNP) for maximizing embryogenesis rate and the number of the somatic embryo per explant, the developed SVR models were exposed to NSGA-II (Fig. 6). Also, a roulette wheel selection method was applied to choose the elite population for crossover [88]. To obtain the best fitness, the initial population, generation number, mutation rate, and crossover rate were respectively adjusted to 200, 1000, 0.5, and 0.7. In the current study, the ideal point of Pareto was selected such that embryogenesis rate and the number of somatic embryos per explant became the maximum. Indeed, a point in the Pareto front was detected as the best optimal answer such that:

11 $$ \sqrt {\left( {embryogenesis \;rate - x} \right)^{2} + \left( {number\; of\;somatic \;embryos\; per\; explant - y} \right)^{2} } $$

Was minimal; where *x* and *y* were the highest embryogenesis rate and the maximum number of somatic embryos per explant in observed data, respectively

### Sensitivity analysis

Sensitivity analysis was conducted to identify the importance degree of KIN, SNP, and 2,4-D on the embryogenesis rate, callogenesis rate, and the number of the somatic embryo per explant. The sensitivity of these parameters was measured by the criteria including variable sensitivity error (VSE) value displaying the performance (root mean square error (RMSE)) of SVR-NSGA-II model when that input variable is removed from the model. Variable sensitivity ratio (VSR) value was determined as the ratio of VSE and SVR-NSGA-II model error (RMSE value) when all input variables are available. A higher important variable in the model was detected by higher VSR.

MATLAB (Matlab, 2010) software was employed to write codes and run the models.

### Validation experiments

In order to approve the efficiency of the developed model, the optimized PGRs (medium containing 9.10 μM 2,4-D, 4.70 μM KIN, and 18.73 μM SNP) obtained from SVR-NSGA-II were experimentally tested in the lab with three replications and each replication consisted of ten leaf explants. The obtained experimental results were compared with predicted results.

## Data Availability

All data generated or analysed during this study are included in this published article.

## References

[1] da Silva JAT (2003). Chrysanthemum: advances in tissue culture, cryopreservation, postharvest technology, genetics and transgenic biotechnology. Biotechnol Adv.

[2] Noda N, Yoshioka S, Kishimoto S, Nakayama M, Douzono M, Tanaka Y, Aida R (2017). Generation of blue chrysanthemums by anthocyanin B-ring hydroxylation and glucosylation and its coloration mechanism. Sci Adv.

[3] Adedeji OS, Naing AH, Kim CK (2020). Protoplast isolation and shoot regeneration from protoplast-derived calli of *Chrysanthemum* cv. White ND. Plant Cell Tissue Organ Cult.

[4] Hesami M, Naderi R, Yoosefzadeh-Najafabadi M (2018). Optimizing sterilization conditions and growth regulator effects on in vitro shoot regeneration through direct organogenesis in *Chenopodium quinoa*. BioTechnologia.

[5] Hesami M, Daneshvar MH (2018). Indirect organogenesis through seedling-derived leaf segments of *Ficus religiosa*-a multipurpose woody medicinal plant. J Crop Sci Biotechnol.

[6] Zimmerman JL (1993). Somatic embryogenesis: a model for early development in higher plants. Plant Cell.

[7] Hesami M, Naderi R, Tohidfar M, Yoosefzadeh-Najafabadi M (2019). Application of adaptive neuro-fuzzy inference system-non-dominated sorting genetic Algorithm-II (ANFIS-NSGAII) for modeling and optimizing somatic embryogenesis of Chrysanthemum. Front Plant Sci.

[8] Mira MM, Wally OS, Elhiti M, El-Shanshory A, Reddy DS, Hill RD, Stasolla C (2016). Jasmonic acid is a downstream component in the modulation of somatic embryogenesis by Arabidopsis Class 2 phytoglobin. J Exp Bot.

[9] Ötvös K, Pasternak TP, Miskolczi P, Domoki M, Dorjgotov D, Szcs A, Bottka S, Dudits D, Fehér A (2005). Nitric oxide is required for, and promotes auxin-mediated activation of, cell division and embryogenic cell formation but does not influence cell cycle progression in alfalfa cell cultures. Plant J.

[10] Zhao Y (2008). The role of local biosynthesis of auxin and cytokinin in plant development. Curr Opin Plant Biol.

[11] Hesami M, Naderi R, Yoosefzadeh-Najafabadi M, Maleki M (2018). In vitro culture as a powerful method for conserving Iranian ornamental geophytes. BioTechnologia.

[12] Jones B, Gunnerås SA, Petersson SV, Tarkowski P, Graham N, May S, Dolezal K, Sandberg G, Ljung K (2010). Cytokinin regulation of auxin synthesis in Arabidopsis involves a homeostatic feedback loop regulated via auxin and cytokinin signal transduction. Plant Cell.

[13] Naing AH, Kim CK, Yun BJ, Jin JY, Lim KB (2013). Primary and secondary somatic embryogenesis in *Chrysanthemum* cv. Euro. Plant Cell Tissue Organ Cult.

[14] Tanaka K, Kanno Y, Kudo S, Suzuki M (2000). Somatic embryogenesis and plant regeneration in chrysanthemum (*Dendranthema grandiflorum* (Ramat.) Kitamura). Plant Cell Rep.

[15] Xu P, Zhang Z, Wang B, Xia X, Jia J (2012). Somatic embryogenesis and plant regeneration in chrysanthemum (Yuukou). Plant Cell, Tissue Organ Cult.

[16] May R, Trigiano R (1991). Somatic embryogenesis and plant regeneration from leaves of *Dendranthema grandiflora*. J Am Soc Hortic Sci.

[17] Hesami M, Daneshvar MH, Yoosefzadeh-Najafabadi M (2018). Establishment of a protocol for in vitro seed germination and callus formation of *Ficus religiosa* L. an important medicinal plant. Jundishapur J Nat Pharm Prod.

[18] Gago J, Landín M, Gallego PP (2010). A neurofuzzy logic approach for modeling plant processes: a practical case of in vitro direct rooting and acclimatization of *Vitis vinifera* L. Plant Sci.

[19] Gürel S, Oğuz MÇ, Turan F, Kazan K, Yüksel Özmen C, Gürel E, Ergül A (2019). Utilization of sucrose during cocultivation positively affects Agrobacterium-mediated transformation efficiency in sugar beet (*Beta vulgaris* L.). Turk J Agric For.

[20] Joung YH, Wu X, Roh MS (2020). Production of high-Quality Ornithogalum thyrsoides cut flowers in one year from in vitro propagated plantlets influenced by plant growth regulators. Sci Hortic.

[21] Guney M (2019). Development of an in vitro micropropagation protocol for Myrobalan 29C rootstock. Turk J Agric For.

[22] Prakash O, Mehrotra S, Krishna A, Mishra BN (2010). A neural network approach for the prediction of in vitro culture parameters for maximum biomass yields in hairy root cultures. J Theor Biol.

[23] Salehi M, Farhadi S, Moieni A, Safaie N, Ahmadi H (2020). Mathematical modeling of growth and paclitaxel biosynthesis in *Corylus avellana* cell culture responding to fungal elicitors using multilayer perceptron-genetic algorithm. Front Plant Sci.

[24] Hesami M, Condori-Apfata JA, Valencia MV, Mohammadi M (2020). Application of Artificial Neural Network for Modeling and Studying In Vitro Genotype-Independent Shoot Regeneration in Wheat. Applied Sciences.

[25] Arab MM, Yadollahi A, Shojaeiyan A, Ahmadi H (2016). Artificial neural network genetic algorithm as powerful tool to predict and optimize in vitro proliferation mineral medium for G × N15 rootstock. Front Plant Sci.

[26] Mridula MR, Nair AS, Kumar KS (2018). Genetic programming based models in plant tissue culture: an addendum to traditional statistical approach. PLoS Comput Biol.

[27] Niazian M, Sadat-Noori SA, Abdipour M (2018). Modeling the seed yield of Ajowan (*Trachyspermum ammi* L.) using artificial neural network and multiple linear regression models. Ind Crops Prod.

[28] Gago J, Martínez-Núñez L, Landín M, Gallego P (2010). Artificial neural networks as an alternative to the traditional statistical methodology in plant research. J Plant Physiol.

[29] Sheikhi A, Mirdehghan SH, Arab MM, Eftekhari M, Ahmadi H, Jamshidi S, Gheysarbigi S (2020). Novel organic-based postharvest sanitizer formulation using Box Behnken design and mathematical modeling approach: a case study of fresh pistachio storage under modified atmosphere packaging. Postharvest Biol Technol.

[30] Nezami-Alanagh E, Garoosi G-A, Landin M, Gallego PP (2018). Combining DOE with neurofuzzy logic for healthy mineral nutrition of pistachio rootstocks in vitro culture. Front Plant Sci.

[31] Arab MM, Yadollahi A, Eftekhari M, Ahmadi H, Akbari M, Khorami SS (2018). Modeling and optimizing a new culture medium for in vitro rooting of G × N15 prunus rootstock using artificial neural network-genetic algorithm. Sci Rep.

[32] Gago J, Pérez-Tornero O, Landín M, Burgos L, Gallego PP (2011). Improving knowledge of plant tissue culture and media formulation by neurofuzzy logic: a practical case of data mining using apricot databases. J Plant Physiol.

[33] Hesami M, Naderi R, Tohidfar M (2019). Modeling and optimizing medium composition for shoot regeneration of chrysanthemum via radial basis function-non-dominated sorting genetic algorithm-II (RBF-NSGAII). Sci Rep.

[34] Jamshidi S, Yadollahi A, Arab MM, Soltani M, Eftekhari M, Sabzalipoor H, Sheikhi A, Shiri J (2019). Combining gene expression programming and genetic algorithm as a powerful hybrid modeling approach for pear rootstocks tissue culture media formulation. Plant Methods.

[35] Hesami M, Naderi R, Tohidfar M (2019). Modeling and optimizing in vitro sterilization of chrysanthemum via multilayer perceptron-non-dominated sorting genetic algorithm-II (MLP-NSGAII). Front Plant Sci.

[36] Ivashchuk OA, Fedorova V, Shcherbinina NV, Maslova EV, Shamraeva E (2018). Microclonal propagation of plant process modeling and optimization of its parameters based on neural network. Drug Invention Today.

[37] Mansouri A, Fadavi A, Mortazavian SMM (2016). An artificial intelligence approach for modeling volume and fresh weight of callus—a case study of cumin (*Cuminum cyminum* L.). J Theor Biol.

[38] Niazian M, Sadat-Noori SA, Abdipour M, Tohidfar M, Mortazavian SMM (2018). Image processing and artificial neural network-based models to measure and predict physical properties of embryogenic callus and number of somatic embryos in ajowan (*Trachyspermum ammi* (L.) Sprague). Vitro Cell Dev Biol Plant.

[39] Munasinghe SP, Somaratne S, Weerakoon SR, Ranasinghe C (2020). Prediction of chemical composition for callus production in *Gyrinops walla* Gaetner through machine learning. Inf Process Agric.

[40] Albiol J, Campmajó C, Casas C, Poch M (1995). Biomass estimation in plant cell cultures: a neural network approach. Biotechnol Prog.

[41] Shiotani S, Fukuda T, Arai F, Takeuchi N, Sasaki K, Kinosita T (1994). Cell recognition by image processing: recognition of dead or living plant cells by neural network. JSME Int J.

[42] Molto E, Harrell RC (1993). Neural network classification of sweet potato embryos.

[43] Zhang C, Timmis R, Hu W-S (1999). A neural network based pattern recognition system for somatic embryos of Douglas fir. Plant Cell Tissue Organ Cult.

[44] Jamshidi S, Yadollahi A, Ahmadi H, Arab M, Eftekhari MJ (2016). Predicting in vitro culture medium macro-nutrients composition for pear rootstocks using regression analysis and neural network models. Front Plant Sci.

[45] Gupta SD, Pattanayak A (2017). Intelligent image analysis (IIA) using artificial neural network (ANN) for non-invasive estimation of chlorophyll content in micropropagated plants of potato. Vitro Cell Dev Biol Plant.

[46] Barone JO (2019). Use of multiple regression analysis and artificial neural networks to model the effect of nitrogen in the organogenesis of *Pinus taeda* L. Plant Cell Tissue Organ Cult.

[47] Niazian M, Shariatpanahi ME, Abdipour M, Oroojloo M (2019). Modeling callus induction and regeneration in an anther culture of tomato (*Lycopersicon esculentum* L.) using image processing and artificial neural network method. Protoplasma.

[48] Mehrotra S, Prakash O, Khan F, Kukreja A (2013). Efficiency of neural network-based combinatorial model predicting optimal culture conditions for maximum biomass yields in hairy root cultures. Plant Cell Rep.

[49] Osama K, Somvanshi P, Pandey AK, Mishra BN (2013). Modelling of nutrient mist reactor for hairy root growth using artificial neural network. Eur J Sci Res.

[50] Araghinejad S, Fayaz N, Hosseini-Moghari S-M (2018). Development of a hybrid data driven model for hydrological estimation. Water Resour Manag.

[51] Fayaz N, Condon LE, Chandler DG (2020). Evaluating the sensitivity of projected reservoir reliability to the choice of climate projection: a case study of bull run Watershed, Portland, Oregon. Water Resour Manag.

[52] Silva JCF, Teixeira RM, Silva FF, Brommonschenkel SH, Fontes EP (2019). Machine learning approaches and their current application in plant molecular biology: a systematic review. Plant Sci.

[53] Vapnik V (1995). The nature of statistical learning theory Springer.

[54] Su Q, Lu W, Du D, Chen F, Niu B, Chou K-C (2017). Prediction of the aquatic toxicity of aromatic compounds to tetrahymena pyriformis through support vector regression. Oncotarget.

[55] Wu C-H, Ho J-M, Lee D-T (2004). Travel-time prediction with support vector regression. IEEE Trans Intell Transp Syst.

[56] Moravej M, Amani P, Hosseini-Moghari S-M (2020). Groundwater level simulation and forecasting using interior search algorithm-least square support vector regression (ISA-LSSVR). Groundwater Sust Dev.

[57] Balabin RM, Lomakina E (2011). Support vector machine regression (SVR/LS-SVM)—an alternative to neural networks (ANN) for analytical chemistry? Comparison of nonlinear methods on near infrared (NIR) spectroscopy data. Analyst.

[58] Sexton J, Everingham Y, Donald D, Staunton S, White R (2018). A comparison of non-linear regression methods for improved on-line near infrared spectroscopic analysis of a sugarcane quality measure. J Near Infrared Spectrosc.

[59] Golkarnarenji G, Naebe M, Badii K, Milani AS, Jazar RN, Khayyam H (2018). Support vector regression modelling and optimization of energy consumption in carbon fiber production line. Comput Chem Eng.

[60] George T, Amudha T (2020). Genetic algorithm based multi-objective optimization framework to solve traveling salesman problem.

[61] Moravej M (2017). Discussion of “Modified Firefly Algorithm for Solving Multireservoir Operation in Continuous and Discrete Domains” by Irene Garousi-Nejad, Omid Bozorg-Haddad, and Hugo A. Loáiciga. J Water Resour Plann Manag.

[62] Srinivas N, Deb K (1994). Muiltiobjective optimization using nondominated sorting in genetic algorithms. Evol Comput.

[63] Hesami M, Daneshvar MH, Yoosefzadeh-Najafabadi M (2019). An efficient in vitro shoot regeneration through direct organogenesis from seedling-derived petiole and leaf segments and acclimatization of *Ficus religiosa*. J For Res.

[64] Goudarzi A, Li Y, Xiang J (2020). A hybrid non-linear time-varying double-weighted particle swarm optimization for solving non-convex combined environmental economic dispatch problem. Appl Soft Comput.

[65] Zielinska S, Kepczynska E (2013). Neural modeling of plant tissue cultures: a review. BioTechnologia.

[66] Akin M, Eyduran SP, Eyduran E, Reed BM (2020). Analysis of macro nutrient related growth responses using multivariate adaptive regression splines. Plant Cell Tissue Organ Cult.

[67] Akin M, Eyduran E, Reed BM (2017). Use of RSM and CHAID data mining algorithm for predicting mineral nutrition of hazelnut. Plant Cell Tissue Organ Cult.

[68] Akin M, Hand C, Eyduran E, Reed BM (2018). Predicting minor nutrient requirements of hazelnut shoot cultures using regression trees. Plant Cell Tissue Organ Cult.

[69] Nezami-Alanagh E, Garoosi G-A, Landin M, Gallego PP (2019). Computer-based tools provide new insight into the key factors that cause physiological disorders of pistachio rootstocks cultured in vitro. Sci Rep.

[70] Khvatkov P, Chernobrovkina M, Okuneva A, Dolgov S (2019). Creation of culture media for efficient duckweeds micropropagation (*Wolffia arrhiza* and *Lemna minor*) using artificial mathematical optimization models. Plant Cell Tissue Organ Cult.

[71] Akbari M, Deligani VJ (2020). Data driven models for compressive strength prediction of concrete at high temperatures. Front Struct Civil Eng.

[72] Arab MM, Yadollahi A, Eftekhari M, Ahmadi H, Akbari M, Khorami SS (2018). Modeling and optimizing a new culture medium for in vitro rooting of G × N15 Prunus rootstock using artificial neural network-genetic algorithm. Sci Rep.

[73] Neill SJ, Desikan R, Hancock JT (2003). Nitric oxide signalling in plants. New Phytol.

[74] Mandal A, Datta S (2005). Direct somatic embryogenesis and plant regeneration from ray florets of chrysanthemum. Biol Plant.

[75] Shinoyama H, Nomura Y, Tsuchiya T, Kazuma T (2004). A simple and efficient method for somatic embryogenesis and plant regeneration from leaves of chrysanthemum [*Dendranthema *×* grandiflorum* (Ramat.) Kitamura]. Plant Biotechnol.

[76] Tymoszuk A, Zalewska M, Lema-Rumińska J (2014). Regeneration of somatic embryos from in vitro isolated ligulate florets of chrysanthemum. Acta Scientiarum Polonorum: Hortorum Cultus.

[77] Leterrier M, Valderrama R, Chaki M, Airaki M, Palma JM, Barroso JB, Corpas FJ (2012). Function of nitric oxide under environmental stress conditions.

[78] Qiao W, Fan LM (2008). Nitric oxide signaling in plant responses to abiotic stresses. Integr Plant Biol.

[79] Laspina N, Groppa M, Tomaro M, Benavides M (2005). Nitric oxide protects sunflower leaves against Cd-induced oxidative stress. Plant Sci.

[80] Hesami M, Tohidfar M, Alizadeh M, Daneshvar MH (2020). Effects of sodium nitroprusside on callus browning of *Ficus religiosa*: an important medicinal plant. J For Res.

[81] Sarropoulou V, Maloupa E (2017). Effect of the NO donor “sodium nitroprusside”(SNP), the ethylene inhibitor “cobalt chloride”(CoCl2) and the antioxidant vitamin E “α-tocopherol” on in vitro shoot proliferation of *Sideritis raeseri* Boiss. & Heldr. subsp. *raeseri*. Plant Cell Tiss Organ Cult.

[82] Rico-Lemus M, Rodríguez-Garay B (2014). SNP as an effective donor of nitric oxide for in vitro plant cell and tissue culture. J Plant Biochem Physiol.

[83] Huang A, She X (2003). Effect of nitroprusside (SNP) on the generation of adventitious roots in mung bean hypocotyl cuttings. Acta Bot Boreal-Occident Sin.

[84] Correa-Aragunde N, Graziano M, Lamattina L (2004). Nitric oxide plays a central role in determining lateral root development in tomato. Planta.

[85] Han X, Yang H, Duan K, Zhang X, Zhao H, You S, Jiang Q (2009). Sodium nitroprusside promotes multiplication and regeneration of *Malus hupehensis* in vitro plantlets. Plant Cell Tiss Organ Cult.

[86] Nolan KE, Irwanto RR, Rose RJ (2003). Auxin up-regulates MtSERK1 expression in both *Medicago truncatula* root-forming and embryogenic cultures. Plant Physiol.

[87] Murashige T, Skoog F (1962). A revised medium for rapid growth and bio assays with tobacco tissue cultures. Physiol Plant.

[88] Mousavi SM, Sadeghi J, Niaki STA, Tavana M (2016). A bi-objective inventory optimization model under inflation and discount using tuned Pareto-based algorithms: NSGA-II, NRGA, and MOPSO. Appl Soft Comput.

